# A Signal Processing Approach with a Smooth Empirical Mode Decomposition to Reveal Hidden Trace of Corrosion in Highly Contaminated Guided Wave Signals for Concrete-Covered Pipes

**DOI:** 10.3390/s17020302

**Published:** 2017-02-07

**Authors:** Javad Rostami, Jingming Chen, Peter W. Tse

**Affiliations:** Department of Systems Engineering and Engineering Management, City University of Hong Kong, Tat Chee Avenue, Kowloon, Hong Kong, China; jmchen1128@gmail.com (J.C.); meptse@cityu.edu.hk (P.W.T.)

**Keywords:** ultrasonic guided wave, NDT, signal processing

## Abstract

Ultrasonic guided waves have been extensively applied for non-destructive testing of plate-like structures particularly pipes in past two decades. In this regard, if a structure has a simple geometry, obtained guided waves’ signals are easy to explain. However, any small degree of complexity in the geometry such as contacting with other materials may cause an extra amount of complication in the interpretation of guided wave signals. The problem deepens if defects have irregular shapes such as natural corrosion. Signal processing techniques that have been proposed for guided wave signals’ analysis are generally good for simple signals obtained in a highly controlled experimental environment. In fact, guided wave signals in a real situation such as the existence of natural corrosion in wall-covered pipes are much more complicated. Considering pipes in residential buildings that pass through concrete walls, in this paper we introduced Smooth Empirical Mode Decomposition (SEMD) to efficiently separate overlapped guided waves. As empirical mode decomposition (EMD) which is a good candidate for analyzing non-stationary signals, suffers from some shortcomings, wavelet transform was adopted in the sifting stage of EMD to improve its outcome in SEMD. However, selection of mother wavelet that suits best for our purpose plays an important role. Since in guided wave inspection, the incident waves are well known and are usually tone-burst signals, we tailored a complex tone-burst signal to be used as our mother wavelet. In the sifting stage of EMD, wavelet de-noising was applied to eliminate unwanted frequency components from each IMF. SEMD greatly enhances the performance of EMD in guided wave analysis for highly contaminated signals. In our experiment on concrete covered pipes with natural corrosion, this method not only separates the concrete wall indication clearly in time domain signal, a natural corrosion with complex geometry that was hidden and located inside the concrete section was successfully exposed.

## 1. Introduction

Pipes carrying gas into residential buildings are critical infrastructure. Inside the buildings, these pipes pass through the concrete walls. To check the integrity of these pipes, a reliable method must be able to test without having access to the entire geometry of the structure. Ultrasonic guided wave is an effective tool to inspect pipes and has been investigated and improved during past years [1,2,3,4,5,6,7]. However, most of the effort in this regard was focused on pipes without any cover or coatings. Existence of coatings around pipes causes limiting inspection range by more attenuation and degrades guided wave test performance. Kwun et al. reported that the coefficients in the coated, buried pipes were two order of magnitude greater for torsional modes in comparison with bare aboveground pipes [8]. Though conducting the test at low frequencies such as 30 KHz could be a solution to such problems, it will limit the guided wave sensitivity to locate the small defects. Barshinger and Rose calculated attenuation dispersion curves for longitudinal modes in hollow cylinder coated with viscoelastic material. They concluded that by changing the mode and frequency of operation, lower attenuation can be achieved [9]. However, using higher frequency can result in excitation of higher order modes that possibly complicate guided wave signals. Predoi described the wave propagation nature in multilayered pipes by means of SAFE method [10]. For buried pipes, depending on the status of coating and mode type, attenuation could be different. Comparing longitudinal L(0,2) with torsional T(0,1), it was demonstrated that for different status of sands, L(0,2) is less attenuative [11,12]. Apart from guided waves in pipes, corrosion in reinforced bar covered by concrete was studied by Miller et al. [13]. Later on Lu et al. [14] extracted statistical parameters for damage detection in rebar-reinforced concrete beams. 

It must be noted that unless a structure and its defect have simple geometries, the interpretation of captured signals may not be straightforward for damage detection. This complication is caused by wave reverberation, the dispersion characteristic of guided waves [15] and the existence of multiple modes within a desired working frequency range [16,17]. Although choosing non-dispersive axisymmetric modes may initially facilitate the test, mode conversion at the location of a non-symmetric defect escalates the complexity of obtained signal [2]. To overcome these difficulties, some studies were focused on developing new sensors for pure mode excitation and efficient transduction of guided waves [18,19,20,21,22]. For example, Vinogradov proposed magnetostrictive transduction systems for generation of torsional modes of guided waves in pipes because of their non-dispersive nature [23]. The new transduction techniques greatly improve the quality of signals in terms of suppressing flexural modes. Being highly dispersive, these flexural modes are mostly preferred to be avoided. The velocities of these modes are normally very close to the excitation mode. This will cause the signal to contain multiple overlapping modes that may cover probable defect indications. It must be noted that, although suppression of unwanted modes is possible in transduction stage, they will appear when guided waves reach non-symmetric and irregular defect shapes.

To obtain more information about guided wave signals, the most straightforward approach is perhaps to utilize one of the Time-Frequency Representation (TRF) methods. Some of the studies during past years were focused on the improvement of different TFRs for guided wave signals with taking their dispersive characteristic into account [24,25,26]. Nevertheless, if the frequency contents of two overlapped wave packages are very close to each other or roughly the same, mode separation by means of conventional TFRs may not be feasible. Chen et al. addressed the corrosion detection in submerged aluminum plate with post processing of the acquired signals [27]. In another attempt for pipes, Tse and Wang [28] estimated the axial length of a defect by matching pursuit. Despite efficiency of matching pursuit, it is computationally expensive and very sensitive to noise. In addition, its performance depends on a dictionary in which dispersion of guided waves is neglected. Matching Pursuit is also too sensitive to the background noise that can deteriorate its outcome. Moreover, Chirplet transform was used to characterize the defects in plates and pipes with artificial notches [29,30]. Being an effective method to separate overlapped modes, Chirplet was applied to a guided wave signals in highly controlled environment without being influenced by real noise. In addition, setting different parameters of Chirplet Transform can add to its computational cost together with its mathematical complexity. 

One of the notable methods for mode separation is the empirical mode decomposition (EMD) that can be applied to wide variety of non-stationary signals [31]. This method effectively separates the overlapping wave packages into intrinsic mode functions (IMFs); however sometimes it may not provide satisfactory results if overlapped modes have the same frequency band. In another word, it may not be able to fully decompose a signal that has multiple overlapping modes. Meanwhile, it suffers from the end effect and redundant IMFs that in some cases might affect the final results [32,33]. Nevertheless, there have been some improvements on EMD method [34,35,36]. Recently, Yan and Lu [37] applied EMD combined with wavelet on a vibration and an electrocardiogram (ECG) signals. In this paper, a similar approach but with a tailored mother wavelet that we call Smooth Empirical Mode Decomposition (SEMD) is adopted to expose a natural corrosion indication hidden in highly overlapped and contaminated signal from a concrete covered pipe. The validation of the proposed method was verified by applying it on different guided wave signals obtained from concrete-covered pipes. To demonstrate the effectiveness of this approach and the amount of improvement it provides, the signals were compared with conventional EMD and Ensemble EMD (EEMD). Meanwhile, for even further validation, we examined the performance of this method by applying it on guided wave signals acquired from field test. Field test data belonged to gas pipes passing through concrete wall in residential buildings.

## 2. Wavelet Transform (WT)

**Figure 1:**
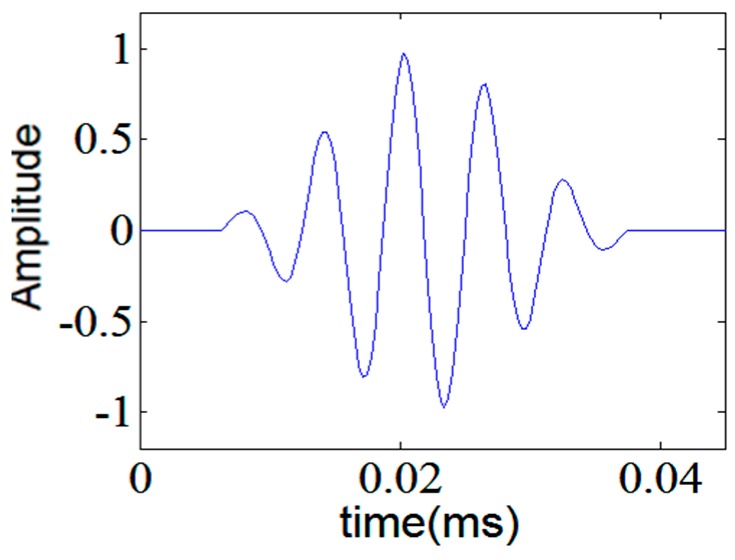
Hamming windowed, five-cycle tone-burst; tailored as a mother wavelet.

The wavelet transform is a powerful tool to assess signals by decomposing them on a set of basic functions. The basis vectors are acquired through a family of functions that depend on scale coefficient and the translation step. It is based on windowing signals into variable sized regions using broader sized windows in time to observe low frequencies, and shorter sized windows in time to spot high frequencies; required by the Heisenberg uncertainty principle [38]. According to Heisenberg principle time-frequency resolution is limited by the following relationship:

(1) $$\sigma_{t}^{2}\sigma_{f}^{2}\geq \frac{1}{4}$$

where $\sigma_{t}^{2}$ and $\sigma_{f}^{2}$ are time and frequency variances, respectively, which represent the local resolution of Wavelet. The Wavelet transform is the sum of the whole signal *x*(*t*) multiplied by a wavelet function (mother wavelet *ψ*(*t*)):

(2) $$W=\frac{1}{\sqrt{s}}\int_{-\infty }^{+\infty }x\left(t\right)\psi \left(\frac{t-l}{s}\right)dt$$

where *s* and *l* are scale and shift, respectively and *W* represents wavelet coefficients. By growing scale, the basis function is dilated; therefore, the corresponding coefficients give information about low frequency components of the signal, and vice versa [39]. It is worthy of emphasis that the success of Wavelet Transform or at least its best result depends on selecting a mother wavelet that maximally matches the shape of the signal that is being analyzed [40]. Considering the right choice of mother wavelet for a particular problem greatly enhances the outcome of the wavelet transform, one can not only choose a mother wavelet among standard wavelet functions but also tailor a function based on prior knowledge about the signal. For example, for guided wave signals a mother wavelet must be chosen or tailored in a way that, it has the maximum resemblance with the excitation signal. For excitation of guided waves in general, narrowband tone-burst signals are used. Tone-burst signal *S*(*t*) is simply a sine wave modulated by windows such as Hamming window:

(3) $$X{\left(t\right)}_{excitation}=\sin\left(\omega t+\theta \right)\left(0.08+0.46\left(1-\cos\left(\frac{\omega t}{N}\right)\right)\right)$$

where *t, ω, θ* and *N* are the time, circular central frequency, phase and number of cycles, respectively. A five-cycle tone-burst signal is depicted in Figure 1.

Among standard wavelet functions Morlet and Gaussian wavelets have a good similarity with the five-cycle tone-burst signal but for different number of cycles in the tone-burst signal, they may not be the best choice. Meanwhile, if this excitation signal is tailored to be used as a mother wavelet, it will give the best results for guided wave analysis. In this paper, a family of analytic tone-burst signals with different number of cycles was designed. Moreover, negative frequency components of each member of the family were discarded with the aid of Hilbert Transform to build analytic wavelet functions. Meanwhile, de-noising can be achieved by wavelet shrinkage and thresholding methods [41]. When thresholding is used for de-noising, it is evident that the threshold level should be higher than the noise level and below the signal level to maintain the critical information of the signal in the first place as well as efficiently remove any contamination. The value of the threshold can be a constant or a function of some well-known parameters in the signals or mother wavelet. For example, for automated inspection systems, the threshold value is advised to be 1.6 to 2.0 times the mean noise level; which means signal to noise ratio must be greater than 4.0 to 6.0 dB. The acoustic noise can result from wave scattering and reverberation of the incident ultrasonic waves that are not related to probable defects. It can be addressed as an uncorrelated Gaussian random variable, with zero mean and a band-limited power spectral density function [40]. Wavelet transform act as filters that decompose the signals into approximations and details. If the details have very low amplitude, they can be removed which consequently decreases noise level without losing critical information. The thresholding is based on discarding all wavelet coefficients whose modulus is smaller than the threshold. The remaining values are subsequently used to reconstruct the signal. 

## 3. The Empirical Mode Decomposition (EMD)

Empirical mode decomposition is an adaptive method introduced to analyze non-linear and non-stationary signals such as guided wave signals. It consists in a local and fully data-driven separation of signals in fast and slow oscillations. EMD works based on iteratively subtract the local mean from the signal to be decomposed to obtain the zero mean components called intrinsic mode functions (IMF). In this method, a signal is decomposed into IMFs that are not necessarily sinusoidal [31]. The summation of all IMFs plus a residue constitutes the original signal *x*(*t*):

(4) $$x\left(t\right)=\sum_{j=1}^{n}IMF_{j}\left(t\right)+r_{n}\left(t\right)$$

Every IMF must satisfy two following conditions:

(1) In the whole dataset, the number of extrema and the number of zero-crossings need to be equal or only differ by one.
(2) At any point, a mean value determined by local maxima envelop and local minima envelop is zero.

Extracting IMFs is an iterative procedure. First, local maxima and local minima in *x*(*t*) are identified and by means of a cubic spline all local maxima and local minima are connected, respectively. After that, mean value of upper and lower envelop *m*_1_(*t*) is calculated and is subtracted from *x*(*t*):

(5) $$h_{1}\left(t\right)=x\left(t\right)-m_{1}\left(t\right)$$

If *h*_1_(*t*) satisfies the two abovementioned conditions, it is the first IMF; otherwise, *x*(*t*) is replaced by *h*_1_(*t*) and the previous process called sifting process is repeated k times until the IMF conditions are satisfied:

(6) h1,j+1(t)=h(t)1,j−m1,j+1(t), j=1, 2,…, k

in which *m*_1,*j+*1_(*t*) is the mean of upper and lower envelop for *h*_1,*j*_(*t*). After finding the first IMF, it is subtracted from the original signal:

(7) $$r_{1}\left(t\right)=x\left(t\right)-IMF_{1}(t)$$

where *r*_1_(*t*) is the residue. Then the residue is treated as new data and the process is repeated to find the remaining IMFs. The process continues until the residue *r_n_*(*t*) becomes too small or a monotonic function from which no more IMFs can be extracted. As EMD suffers from mode mixing problem, it is further on developed into Ensemble Empirical Mode Decomposition (EEMD) by Wu and Huang [42] to alleviate this shortcoming. In the EEMD method, a white noise is initially added to the signal and decomposing the signal into IMFs is carried out afterwards; similar to EMD. As the white noise is random, the process is repeated over a large number with different sets of white noise. The final decomposition result is acquired by finding the mean of each IMF from each signal decomposition process. The amplitude of white noise substantially affects the performance of EEMD so that if it is too small, it may not prevent mode mixing and if it is too large, the number of ensemble averages required might be too high to suppress the noise in the ensemble averaged IMFs. The value is suggested to be 0.1–0.4 of the standard deviation of the original signal.

## 4. Smooth Empirical Mode Decomposition (SEMD)

Being an effective tool to reveal physical meanings of a signal, EMD suffers from multiple shortcomings that were earlier mentioned. One of its major problems is the frequent appearance of mode mixing. Mode mixing means that an IMF consists of signals of widely disparate scales or signals of a similar scales residing in different IMF components. EEMD provide improvement for mode mixing when the analyzed signal contains high frequency intermittent oscillations [43]. Meanwhile, EMD experiences end effects problems and redundant IMFs that in some cases might affect the final results [42]. Yan and Lu [37] presented an improvement on Hilbert-Huang transform to solve the end effect problem for ECG signals. End effects issue is caused as a result of the lack in a number of extrema at both ends of a signal. This lack deteriorates spline curve fitting process and consequently corrupts each IMF with complex frequency components. To reduce this effect, in SEMD the wavelet transform clarified in the earlier section is applied on IMFs during sifting the process by the following equation:

(8) $$W_{j,k}=\int_{-\infty }^{+\infty }IMF_{i}\left(t\right)\psi_{j,k}\left(t\right)dt$$

where *W_j,k_* is the *k*th wavelet coefficient at the *j*th level, *j*, *k* ϵ Z. And *ψ_j,k_*(*t*) is the mother wavelet. Threshold de-noising is then utilized to eliminate unwanted components in every IMF. After de-noising, new wavelet coefficients are estimated and subsequently a new IMF is reconstructed. After that, unlike conventional EMD, this time, the new purified IMF′ is subtracted from the original signal:

(9) $$r_{i}\left(t\right)=x\left(t\right)-IM{F^{\prime }}_{i}(t)$$

The abovementioned process is repeated in every sifting stage so that new IMFs are extracted from the signal. As previously mentioned, for guided wave signals, incident waves are well known and consequently it can be inferred that the signals consist of a combination of the incident waves plus unwanted reverberations. The same assumption can be made for IMFs that consequently form the original signal. Therefore, for guided wave signals in which the tone-burst signal is selected for the excitation, this assumption can be made when dispersion characteristic of guided waves is neglected. As a result, instead of using standard well-known mother wavelets, in SEMD we can tailor a complex tone-burst function as a mother wavelet which best matches the ultrasonic guided wave signal. The steps for implementing SEMD and finding *IMF_i_*’ are as follows:

(1) Compute *IMF_i_* according to the procedure discussed in Section 3.
(2) Calculate the wavelet coefficients according to the equation described in Section 2 based on tone-burst wavelet:
  $$W=\frac{1}{\sqrt{s}}\int_{-\infty }^{+\infty }IMF\left(t\right)\psi \left(\frac{t-l}{s}\right)dt$$
(3) Estimate de-noised new wavelet coefficients by predefined positive value of threshold *T*:
  $$d\left(W\right)=\mathrm{sgn}\left(W\right){\left(\left|W\right|-T\right)}_{+}$$
(4) Reconstruct *IMF_i_’* from the de-noised coefficients through the inverse wavelet transform.
(5) Subtract *IMF_i_’* from *x*(*t*) and obtain the residue *r_i_*(*t*)

Replace *x*(*t*) with the residue and return to step 1; repeat the iteration process and continue until the residue *r_n_*(*t*) becomes too small or a monotonic function from which no more *IMFs* can be extracted. It is worthy of mention that this method is different than some works with a similar names reported in [44,45,46,47].

## 5. Experimental Validations

### 5.1. Experimental Setup

**Figure 2:**
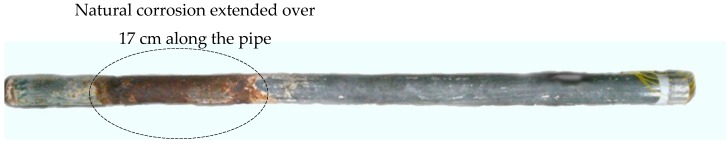
Steel pipe with natural corrosion.

To verify the applicability of the proposed method experiments were carried out in two stages including a laboratory test and a field test. In Stage 1, the experiment was conducted on two 80 cm long steel pipes. They were standard pipes delivering natural gas to residential buildings with the diameter of 33 mm and 4 mm wall thickness. One of them was pristine while the other had a corrosion extended over 17 cm along the pipe. The corrosions were naturally formed and the pipes were provided by the sole gas supplier in Hong Kong (Figure 2). 

**Figure 3:**
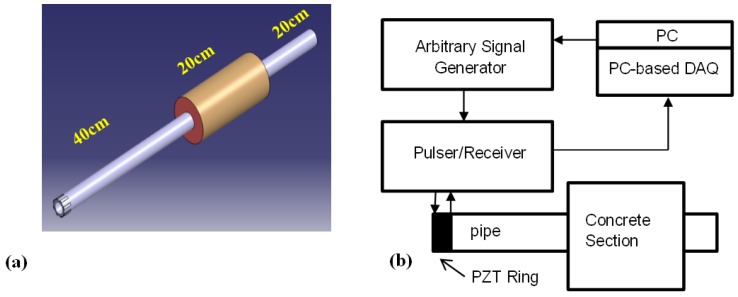
Concrete covered pipe (**a**); and schematic diagram of experimental setup; concrete covered pipe (**b**).

**Figure 4:**
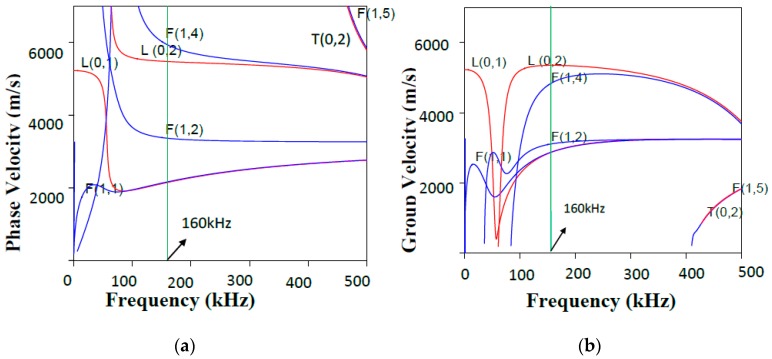
Phase (**a**); and group (**b**) velocity dispersion curves of the examined pipe.

**Figure 5:**
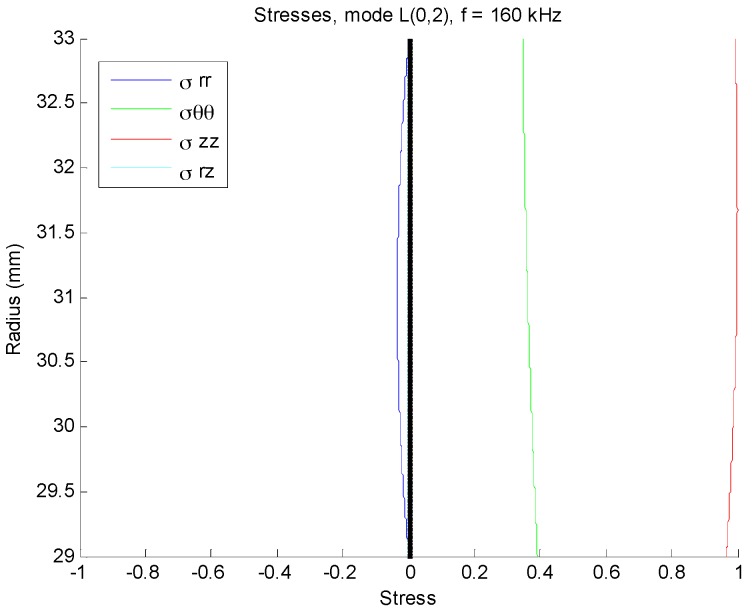
Stress field of L(0,2) mode at 160 kHz in a steel pipe.

Both pipes were partially covered by concrete to simulate real concrete walls at residential buildings (Figure 3a). For the corroded pipe, the defective part was placed inside the 20 cm long concrete wall. Figure 3b illustrates a schematic diagram of our experimental setup. For the corroded pipe the concrete wall is located 48 cm away from one end which is 8 cm different from the normal pipe. An array of PZT strips was bonded at one end of the pipes to transmit and receive guide waves in pulse-echo mode. The strips are axisymmetrically distributed, so that longitudinal modes excitation is guaranteed and flexural modes are suppressed. L(0,2) mode in 160 kHz was preferred over the other guided wave modes because of its straightforward excitation and its less attenuation in comparison with torsional mode T(0,1) [11]. Lower attenuation, makes L(0,2) mode more feasible to inspect pipes buried in sand or covered by concrete. In this frequency, it is non-dispersive with the highest group velocity as depicted in the dispersion curves in Figure 4. PCdisp open source MATLAB toolbox which is based on Pochhammer-Chree dispersion equation is used to plot dispersion curves [48,49]. Meanwhile, with the aid of this toolbox we can observe that this mode has uniform stress distribution over the pipe wall thickness. Figure 5 demonstrates the stress filed of L(0,2) at 160 kHz. The uniform stress distribution makes the L(0,2) equally sensitive to any change; regardless of their radial positions in the pipes’ wall thicknesses. For the excitation of L(0,2) mode at the selected frequency five-cycle tone-burst signal modulated by Hamming window was delivered through an arbitrary signal generator and generated by an RITEC 4000 pulser & receiver (RITEC Inc., Warwick, RI, USA):

(10) $$X{\left(t\right)}_{excitation}=\sin\left(\omega t+\theta \right)\left(0.08+0.46\left(1-\cos\left(\frac{\omega t}{5}\right)\right)\right)$$

**Figure 6:**
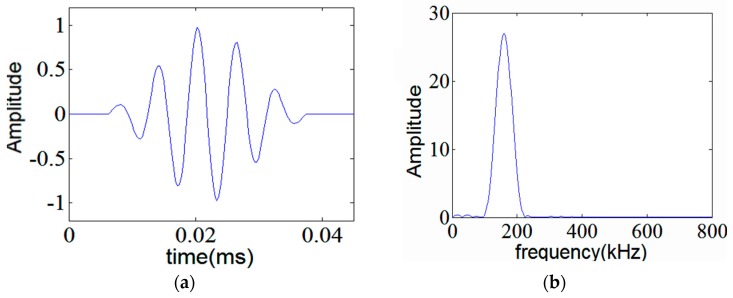
Five-cycle tone-burst at 160 kHz center frequency (**a**) and its spectrum (**b**).

The frequency band of tone-burst signal can be easily changed by altering the number of cycles. By increasing the number of cycles in the tone-burst signals, the narrower band signals can be obtained. Figure 6 shows the excitation signal together with its frequency spectrum.

**Figure 7:**
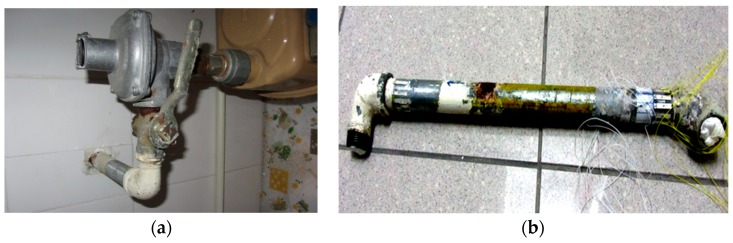
The in-service pipe used for the field test: the indoor portion of the pipe with rust starting at the entry of the concrete wall (**a**); and the corroded pipe with rusts after dissembled it from the concrete wall (**b**).

**Figure 8:**
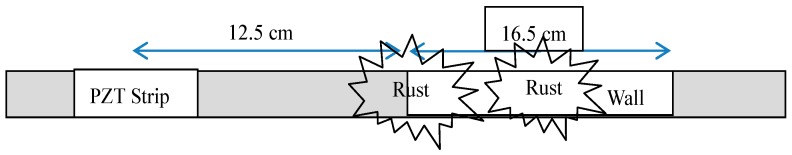
Schematic diagram of experimental setup for on-site testing of pipes carrying gas to residential buildings.

In Stage 2, we repeated the experiment on pipes carrying gas to residential buildings in Hong Kong. Similar to the experiment conducted in our lab, a strip of PZTs was mounted on a pipe that was passing through a concrete wall (Figure 7). For the pipe with corrosion, rusty portion was started at the entry of the concrete wall and extended all the way to the portion of pipe that was covered by the concrete wall. However, there were two areas in which the corrosion was severe: the entry of the concrete wall and an area right before the end of the concrete wall. Figure 8 demonstrates the schematic diagram of our setup with the location of most severe part of the corrosion on the pipe. After completing data collection, the in-service corroded pipe was replaced with a new and normal pipe. The data collection process was repeated again on this normal pipe for comparison purpose.

### 5.2. Results and Discussion

After obtaining the guided wave signals for both pipes, in this section, our goal was focused on two main points. First, the location of concrete on the both signals must be identified. In other words, the signs reflecting the existence of concrete and its position in the signals must be determined. The second point, which is the most important one is to answer the question of whether there is corrosion in the pipe and if yes at what location. The prerequisite for such goals is to identify the group velocity value by which time of flights for relevant wave packages are calculated. The group velocity (*v_g_*) can be pinpointed on the dispersion curves in Figure 4. As an alternative, group velocity can be calculated by measuring time of flights on a signal that belongs to a normal pipe:

(11) $$v_{g}=\frac{L}{D}$$

in which *L* is the twice length of the normal pipe prior covering it with concrete in pulse echo-mode and *D* is the time shift between near and far end signals. The reason for selecting a normal pipe without concrete cover for the calculation is the simplicity of guided wave signals without any overlapping modes. Parameter *D* can be manually measured but to accurately determine *D*, cross correlation function (CCF) of the two signals of pipe ends is computed. The time shift between the two signals corresponds to the maximum value of their CCF [50] as shown below:

(12) $$D=\max\left(\frac{1}{T}\int_{0}^{T}x\left(t\right)y\left(t+\tau \right)dt\right)$$

where *T* is observation time, and *x* and *y* are near and far end signals, respectively. The group velocity for our experiment was determined to be 5100 m/s. It is worthy of mention that *v_g_* can be calculated in pitch-catch mode as well; if we install another strip of PZT in a distance *L* from the first strip. 

Table 1 lists the different time of flights that indicate critical positions in the pipes. In other words, we expect to see those positions at calculated time of flights according to Table 1. The time values in Table 1 were calculated by dividing the traveling distance by 5100 m/s which is the group velocity.

**Figure 9:**
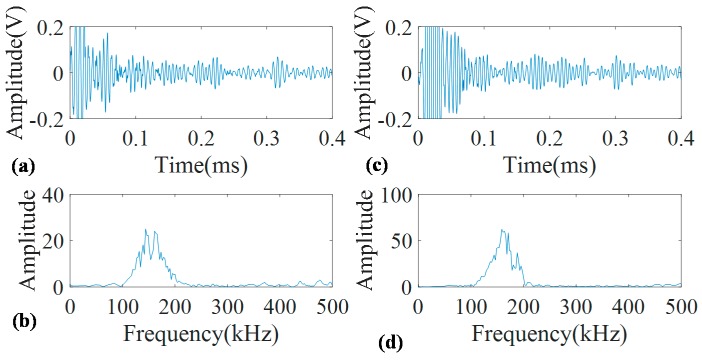
Guided wave signals measured at 160 kHz for concrete covered pristine pipe (**a**) with its FFT (**b**); and concrete covered corroded pipe (**c**) with its FFT (**d**).

Figure 9 demonstrates the signals obtained from the concrete covered pipes. As can be seen, the time domain signals are too complicated to locate concrete section and the corrosion. Although the critical positions were identified according to Table 1, distinguishing them in the signals in Figure 9 is not straightforward and almost impossible. Meanwhile, looking at the frequency spectrum of both signals, one can see that they are distorted from the original FFT in Figure 6b and they contain some other frequency components. Comparing these two signals, the signal of the corroded pipe has higher energy reflected from the concrete section. Having higher reflection energy although could be a sign of corrosion; it may not enough to assure such conclusion. This is because rough surface of the rusty part in concrete wall facilities the coupling of two materials so that they are strongly bonded together and signal energy eventually may fade away inside the concrete wall without having much of it left to reflect back. Meanwhile, in real situation since the walls have very big lengths, we may not see the reflection of guided waves once they leak into the concrete. Such issues dictate the necessity of using an advanced signal processing technique to provide an insight into the real situation of the pipes.

**Figure 10:**
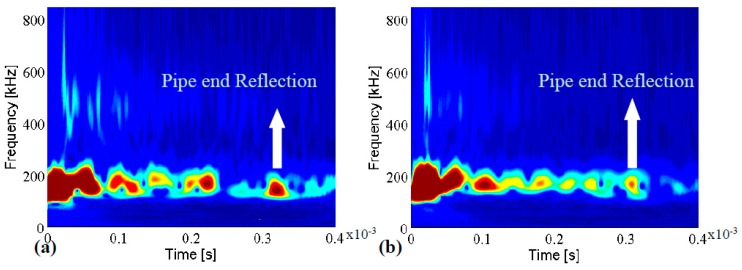
Continuous wavelets transform of the pristine pipe (**a**); and the pipe with corrosion (**b**).

To find the localized frequency components of the signals, wavelet was used to draw their time-frequency representations (TFRs). Figure 10 demonstrates continuous wavelet transform of the signals in Figure 9 by adopting complex tone-burst wavelet. As can be seen in Figure 10, the two TFRs look very similar and not only cannot we locate the concrete wall but there is large amount of ambiguity about the existence of the defect. This problem occurs because of many overlapping modes coming with the environmental contamination. Nevertheless, despite the difficulty in extracting the critical information, detecting the pipe ends at 0.31 ms is possible. The simplicity in detecting the end is because of the fact that the end acts as a block that reflects all the energy back towards the incident point. 

**Figure 11:**
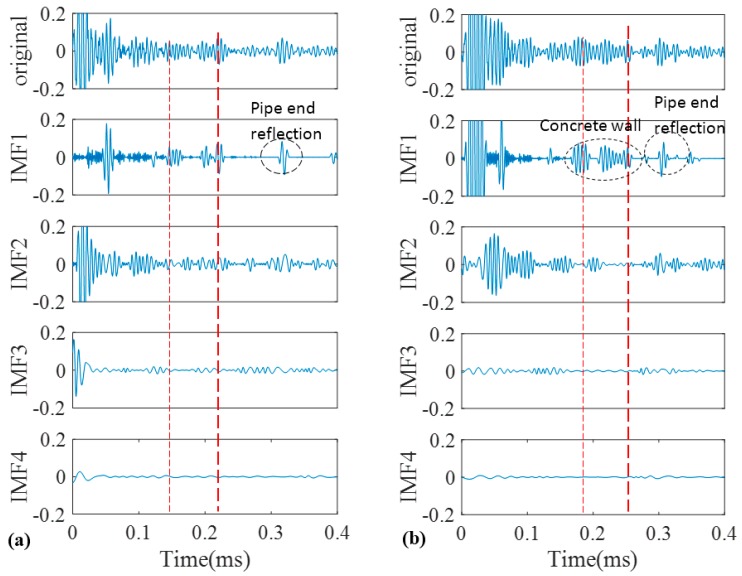
Decomposition of signals into their IMFs by EMD for the pristine pipe (**a**); and the pipe with a natural corrosion (**b**).

As a good candidate to decompose a non-stationary signal into its intrinsic mode functions, we firstly adopted conventional EMD for our recorded signals. The signals obtained in Figure 9 were decomposed into different IMFs according to the algorithm that was explained in the earlier section. The original signals of the pristine and corroded pipes together with their IMFs are demonstrated in Figure 11a,b, respectively. Two vertical dashed lines passing through all IMFs indicate the location of concrete wall on time axis according to Table 1.

Only first four IMFs are illustrated in Figure 11. The higher order IMFs, due to having very low energy and frequency inconsistency with the excitation frequency band are neglected. To find which IMF contains the most relevant information, cross correlation of the original signals and their IMFs were calculated. Cross correlation is a tool of measuring the similarity between two signals which in our case are an original guided wave signal *x*(*t*) and a corresponding IMF [51,52]:

(13) $$R\left(\tau \right)=\frac{1}{T}\int_{0}^{T}x\left(t\right)IMF\left(t+\tau \right)dt$$

**Figure 12:**
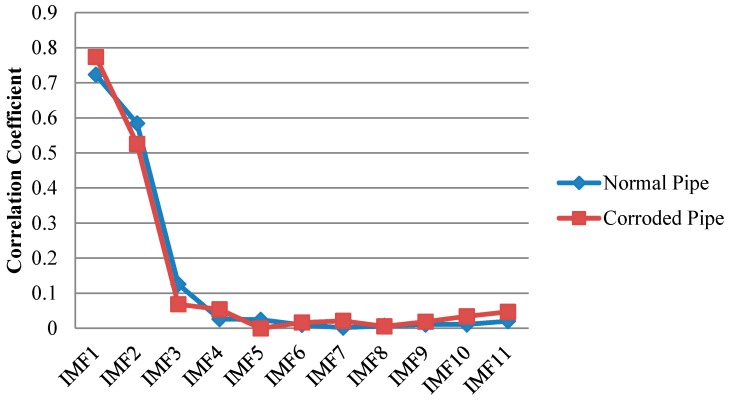
Absolute cross correlation of guided wave signals from pipes with their corresponding IMFs obtained from EMD.

The normalized value of cross-correlation is a number between −1 and 1. The bigger absolute value of cross-correlation, indicate more similarity between two signals (Figure 12).

**Figure 13:**
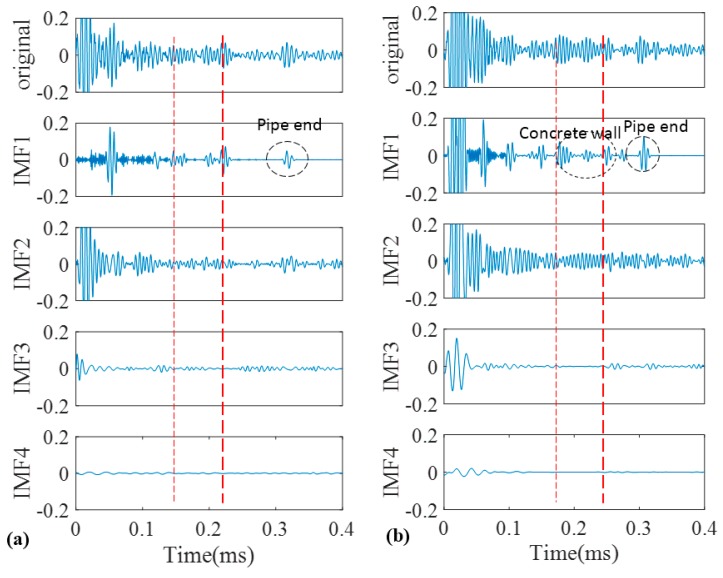
Decomposition of signals into their IMFs by EEMD for the pristine pipe (**a**); and the pipe with a natural corrosion (**b**).

As illustrated in Figure 11 we may only partially achieve our goals in exposing critical features of the pipes. First two IMFs have the greatest portion of the signals’ energy. Meanwhile they both have the maximum similarity with the original signal as their absolute values for cross-correlation are much bigger than other IMFs (Figure 12) However IMF1 has the largest absolute correlation and therefore, is of more importance. We take the absolute cross-correlation as a criterion to select an IMF for further analysis. The effect of other IMFs on physical meaning of the original signal is neglected because of the lower values for cross-correlation. Now with the aid of EMD positions of the ends are very evident especially for the pristine pipe as exposed in IMF1 for the both pipes. It must be mentioned that the pristine pipe is slightly longer and as a result the location of pipe end reflection happens with a few microseconds delay. Apart from that, for the concrete section, one can see that it is almost visible in IMF1 for both pipes; although IMF1s are highly noisy and contaminated. This occurrence is because of signal reverberation and generation of unwanted modes and contamination when guided waves reach the concrete wall. Therefore, both groups of IMFs include other reverberation signals which might be difficult to interpret. Being better than EMD, EEMD was later applied on the same signals in Figure 9. The results obtained from EEMD are depicted in Figure 13.

**Figure 14:**
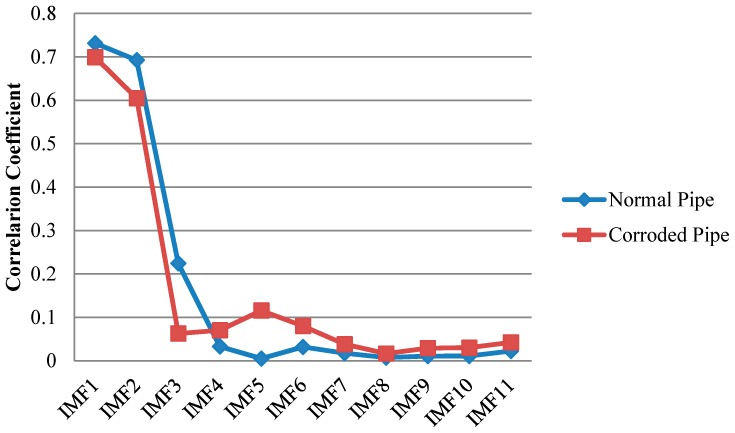
Absolute cross correlation of guided wave signals from pipes with their corresponding IMFs obtained from EEMD.

Similar to EMD, cross correlation was used to find which IMFs for both pipes contain the critical information. As can be observed in Figure 13, comparable to the results of EMD, IMF1s include much information about the critical features of the pipes as their features can be somehow spotted in the first IMFs. This is in an agreement with the correlation results in Figure 14 which means IMF1s have the most relevant embedded information. The reflections from the far end for both pipes are exposed in IMF1s. In addition, while the concrete wall is separated for the corroded pipe, in the normal pipe IMF1 still have many overlapped components. Meanwhile, for corrosion detection, comparison study between the second IMFs does not provide useful and clear information. 

**Figure 15:**
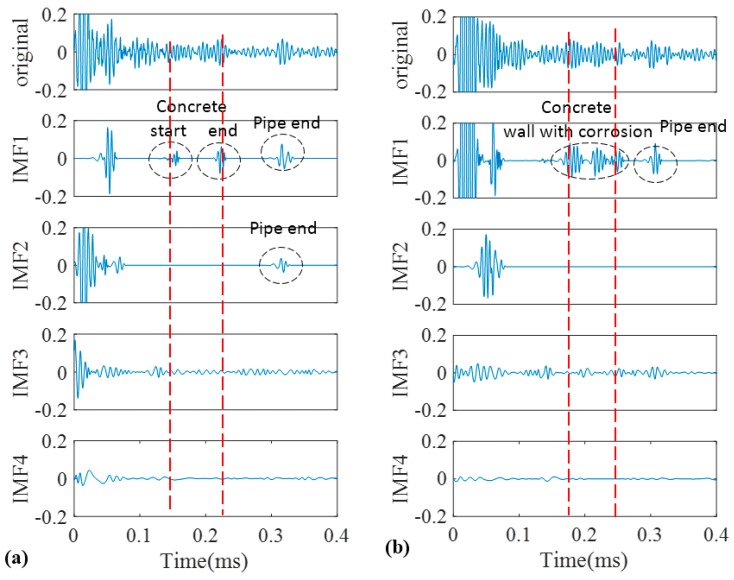
Decomposition of signals into their IMFs by SEMD for the pristine pipe (**a**); and the pipe with a natural corrosion (**b**).

**Figure 16:**
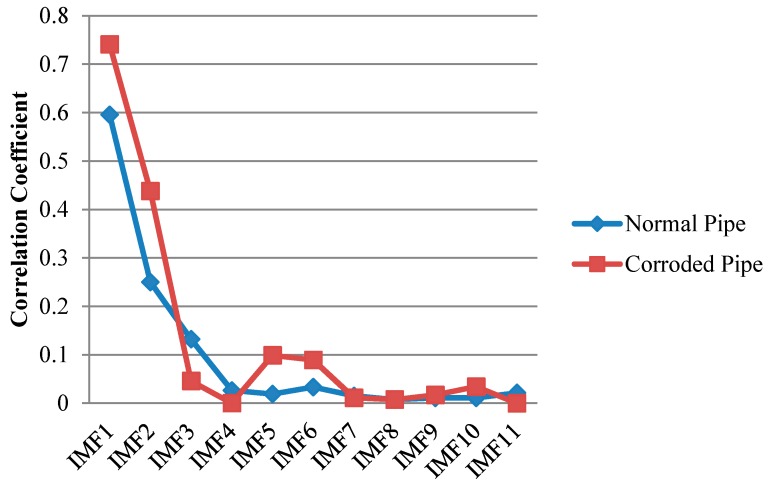
Absolute cross correlation of guided wave signals from pipes with their corresponding IMFs obtained from SEMD.

Furthermore, to overcome the shortcomings of conventional EMD, we utilized the SEMD whose theory was already discussed in a former section in this paper. The main point is that not only guided wave signals may contain many contaminations; each IMF may also include some unwanted components. Thus, with the aid of wavelet transform on IMFs in sifting process unwanted reverberations can be discarded from their wavelet coefficients. For wavelet transform, complex tone-burst wavelet was used as a mother wavelet whose design was already explained. Figure 15 demonstrates the signal from the pristine and corroded pipe together with their IMFs. Similar to previous results, in SEMD only four IMFs are depicted as the rest have very low energy in comparison with the original signal. As is clear in Figure 16, IMF1s have the most relevant information about the original signals for both the normal pipe and pipe with corrosion. Hence, the first IMFs were taken into account for further analysis. Selecting first IMFs can be verified through the comparison of the correlation of IMFs and original signals in Figure 16. 

The first IMFs have the maximum similarity with the original signal because of their biggest values of absolute cross correlation in Figure 16. As can be seen, all critical information about this pipe is clearly exposed in IMF1. The locations of concrete wall plus the pipe end in time axis can be easily verified according to Table 1. Since the reflection of guided wave from the end has much higher energy, it can be observed in IMF2 as well. In IMF1 between the start and end of concrete wall, signal is very clear and there is no implication of any defect. It should be noted that the first wave packet in IMF1 which can also be seen in the original signal, is the reflection from the position of a PZT ring that had initially been installed there to measure the group velocity in pitch-catch mode. In addition, the first wave packet in IMF2 is the reflection of guided wave from the near end (close to PZT ring in Figure 3). Moreover, Figure 15b demonstrates the signal for the corroded pipe in conjunction with its IMFs. Similar to the first IMF for the pristine pipe, location of the concrete wall and the pipe end are evident in the IMF1 and can be verified again according to Table 1. Nonetheless, inside the concrete wall the signal is chaotic with many reverberations. This complexity in this part of the signal indicates the natural corrosion with an irregular geometry that is extending over 17 cm in the pipe (Figure 2). Meanwhile, as it was mentioned earlier, all the corrosion was placed inside the concrete wall. Therefore, seeing such wave packets inside the wall is not unexpected. In addition, unlike the IMF2 for pristine pipe, the reflection from the pipe end does not appear in the second IMF. This is because much of signal energy was already reflected back towards the PZT ring as a result of the guided wave hitting the corrosion. The higher order IMFs for both the pristine pipe and the corroded pipe do not have meaningful information as the signal energy becomes very low and their absolute cross correlation is close to zero.

**Figure 17:**
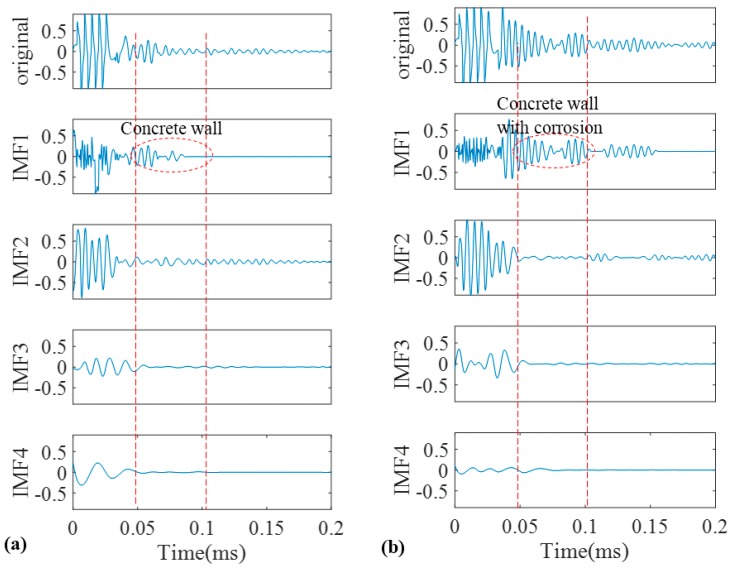
Decomposition of field test signals into their IMFs by EMD for the pristine pipe (**a**); and the pipe with a natural corrosion (**b**).

The data obtained in the Stage 2 from normal and concrete-wall covered pipes in the field test, as per Stage 1, the conventional EMD, EEMD and finally SEMD were used for the signal analysis. Figure 17 depicts the original signals obtained from normal and rusty pipe in field test together with their IMFs from EMD.

**Figure 18:**
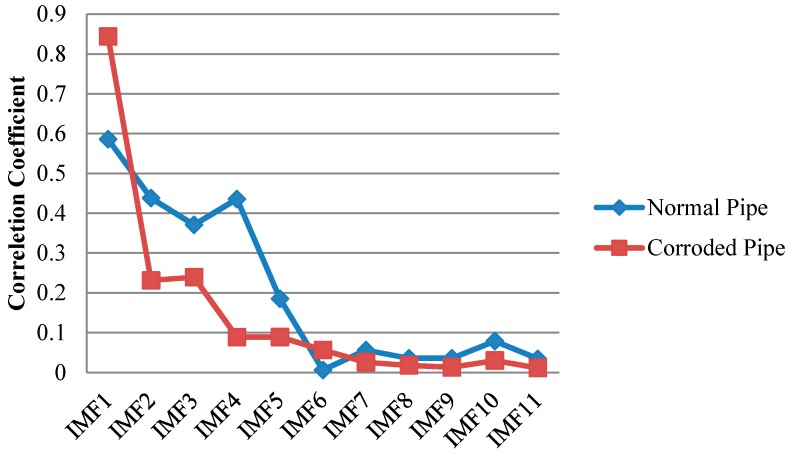
Absolute cross correlation of guided wave signals from the pipes in residential building with their corresponding IMFs obtained from EMD.

Although because of the severe corrosion, the higher reflection energy of the original signals in Figure 17 can address a defect, the signals are highly contaminated that could be taken as source of ambiguity. Cross correlation of IMFs of the EMD with the original signals are demonstrated in Figure 18. In IMF1 for the pristine pipe, within the concrete wall there are two main wave-packets. While the first one could be taken as the sign of the beginning of the concrete wall, the existence of the second wave-packet remains unanswered. For the pipe with the two corrosion areas, IMF1 shows larger energy reflecting back from the concrete wall. However, it is not much different from the original signal. 

**Figure 19:**
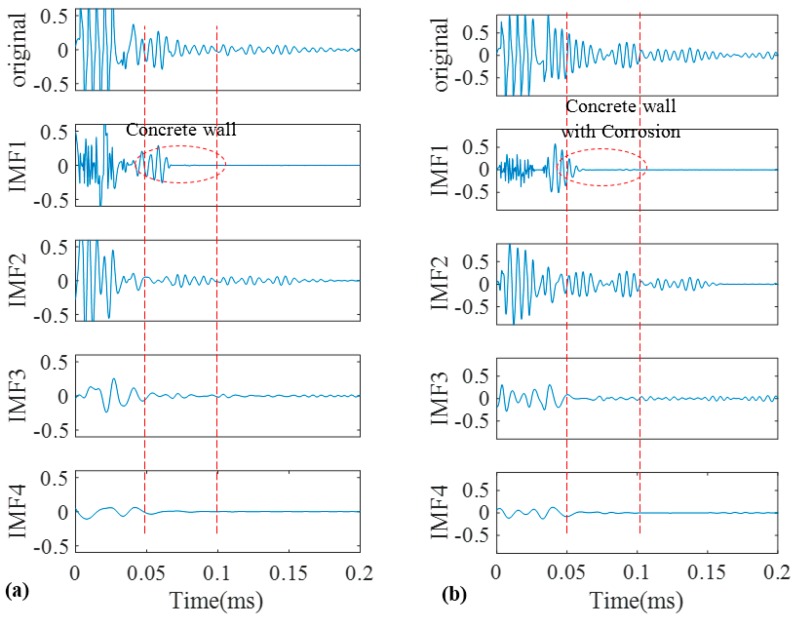
Decomposition of field test signals into their IMFs by EEMD for the pristine pipe (**a**); and the pipe with a natural corrosion (**b**).

**Figure 20:**
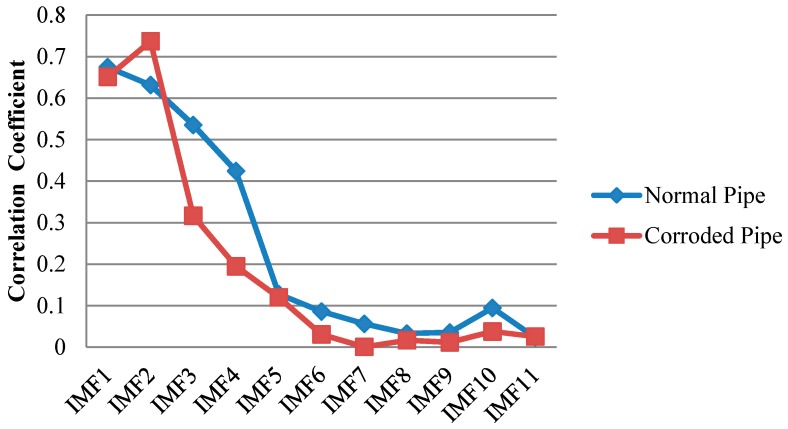
Absolute cross correlation of guided wave signals from the pipes in residential building with their corresponding IMFs obtained from EEMD.

Figure 19 illustrates the same signals decomposed by EEMD. Similar to the previous sections only four IMFs are shown in the figure. The remaining IMFs whose energies approach to zero were neglected. Cross correlations of the IMFs with the original signals are depicted in Figure 19. For the pristine pipe, IMF1 has the maximum similarity according to Figure 19. Compared to IMF1 from EMD, EEMD provides a better result as there is only one wave-packet in the beginning of the concrete wall that indicates the start point of the wall. For the corroded pipe, unlike the normal pipe, IMF2 has the maximum correlation coefficient and as can be seen in Figure 20 is full of mode reverberations very similar to the original signal. Meanwhile, looking at the results from EMD and EEMD, the concrete wall cannot be separated from the rest of the signal as it was clearer in our experiment in Stage 1 in the lab. 

**Figure 21:**
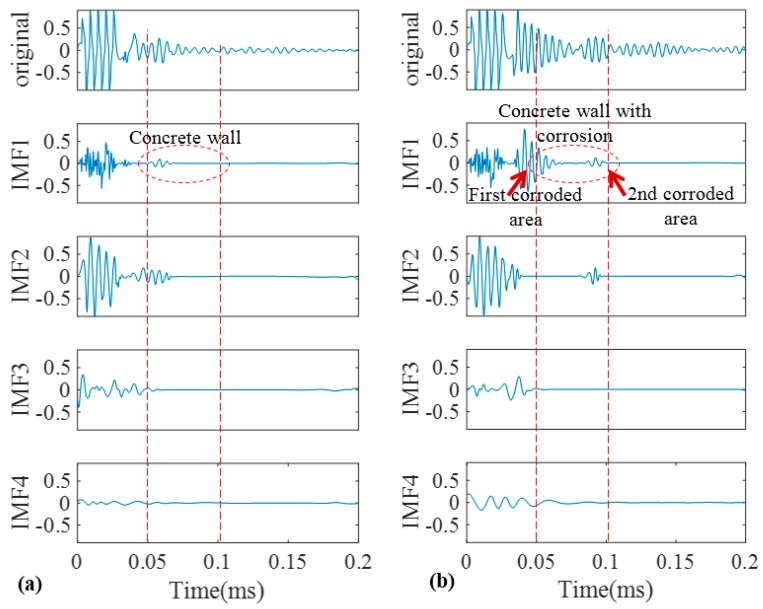
Decomposition of signals into their IMFs by SEMD for the pristine pipe (**a**); and the pipe with a natural corrosion (**b**).

In our next step, we applied SEMD to acquire much clearer and easier to interpret results. The original signal from our field test together with IMFs obtained by SEMD are shown in Figure 21. As one can see from this figure, unlike the results in the lab, we do not have pipe end as the pipes connected to a network with elbows. Therefore, since there is no blockage for wave propagation as well as high attenuation within the concrete wall section, it is very difficult to locate the pipe elbows. According to the calculation of time of flight for L(0,2) mode that propagates with 5100 m/s velocity and considering the distance of concrete wall from PZT strips in Figure 8, we expect to see the beginning and end of the concrete wall at 0.49 and 1.11 ms. However, unlike the signals in obtained in our lab, the end of the concrete wall remains hidden. This is because, in field test the wall has infinite length and when guided waves propagate and leaks into the wall, it attenuates inside the wall and we cannot see its reflection. Nevertheless, the two corrosion spots were clearly extracted from the signal and can be easily located in IMF1.

**Figure 22:**
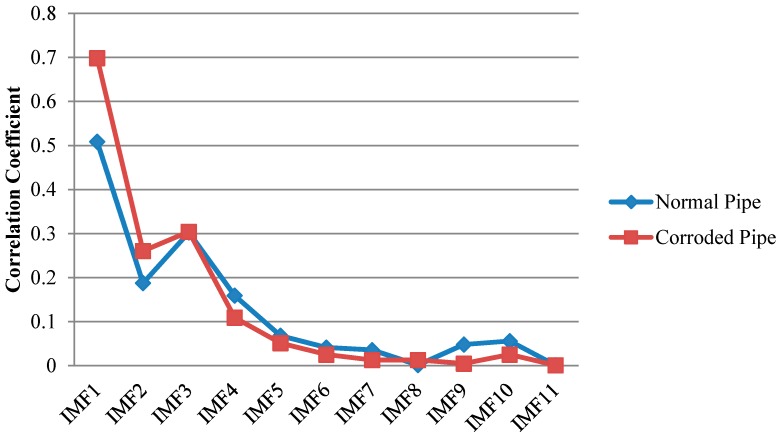
Absolute cross correlation of guided wave signals from the pipes in residential building with their corresponding IMFs obtained from SEMD.

Analogous to SEMD result from the lab test, the first IMF has the most relevant information about two pipes. This can be proved by comparing absolute values of cross correlation of the IMFs with the original signals. As can be seen in Figure 22, for the two signals, IMF1 has the largest value among the other IMFs meaning it has maximum similarity with the original signal. In other words, Figure 22 provides a clue about the most important IMFs to be taken into consideration.

## 6. Conclusions

In this paper we focused on the separation of overlapped modes in concrete-wall covered pipes using a new signal processing technique. The study was carried out on a pristine pipe and a pipe with a natural corrosion. A PZT ring was used to excite and receive guided waves in pulse-echo mode and both pipes were partially covered by concrete. Guided wave signals obtained in such pipes were very complicated because of the existence of the overlapped modes and wave reverberations. Initially wavelet transform was used to study localized frequency components in the signal. Afterwards, conventional EMD and later EEMD were applied on the signals. The two methods did not perform well for interpretation of the signals, and the best results were obtained utilizing SEMD which is the integration of the tailored wavelet and EMD. Cross Correlation was used to select the IMF best matches with the original signals. For SEMD, a novel mother wavelet was tailored based on the information about incident waves which was the tone-burst signal. Wavelet transform then was used to purify the IMFs in the sifting process. This is because not only raw signals are contaminated with unwanted components and wave reverberations, their IMFs may also suffer from the same issue. Thus, wavelet de-noising tool substantially enhanced the performance of this signal processing technique for guided waves in wall covered pipes. The positions of the concrete wall, pipe end and defect in time axis were distinctly exposed. These locations were verified by calculating the time of flight of L(0,2) mode traveling the well-known distances on our pipes. The defect signal was not a single wave packet and consisted of multiple small waves. This was because of irregular shape of corrosion and its extension inside the concrete wall. For further validation, the experiment conducted in a residential building on pipes carrying gas. The irregular shape corrosion was successfully revealed and separated from the rest of the signal and confirmed the additivity of this method for applying highly contaminated guided wave signals.

## Figures and Tables

**Table 1:** Calculated time of flights for critical positions in the concrete-covered pipes.

Group Velocity 5100 (m/s)	Time of Flight (ms)		
	t_1_ (Start of Concrete Wall)	t_2_ (End of Concrete Wall)	t_4_ (Pipe End)
Normal Pipe	0.15	0.23	0.31
Pipe with Corrosion	0.18	0.26	0.31
